# Integrated Optic Surface Plasmon Resonance Measurements in a Borosilicate Glass Substrate

**DOI:** 10.3390/s8117113

**Published:** 2008-11-11

**Authors:** Antonino Parisi, Alfonso C. Cino, Alessandro C. Busacca, Matteo Cherchi, Stefano Riva-Sanseverino

**Affiliations:** 1 C.R.E.S., Centro per la Ricerca Elettronica in Sicilia, Via Regione Siciliana 49, 90046 Monreale (PA), Italy; E-mails: parisi@cres.it; prescres@cres.it; 2 DIEET, Dipartimento Ingegneria Elettrica, Elettronica e delle TLC, Università di Palermo, Viale delle Scienze, 90128 Palermo, Italy; E-mails: cherchi@unipa.it (M.C.); busacca@unipa.it (A.C.B.)

**Keywords:** Biosensors, Integrated Optics, Surface Plasmon Resonance

## Abstract

The surface plasmon resonance (SPR) technique is a well-known optical method that can be used to measure the refractive index of organic nano-layers adsorbed on a thin metal film. Although there are many configurations for measuring biomolecular interactions, SPR-based techniques play a central role in many current biosensing experiments, since they are the most suited for sensitive and quantitative kinetic measurements. Here we give some results from the analysis and numerical elaboration of SPR data from integrated optics experiments in a particular borosilicate glass, chosen for its composition offering the rather low refractive index of 1.4701 at 633 nm wavelength. These data regard the flow over the sensing region (metal window) of different solutions with refractive indexes in the range of interest (1.3÷1.5) for the detection of contaminants in aqueous solutions. After a discussion of the principles of SPR, of the metal window design optimization by means of optical interaction numerical modeling, and of waveguide fabrication techniques, we give a description of system setup and experimental results. Optimum gold film window thickness and width in this guided-wave configuration has been for the first time derived and implemented on an integrated optic prototype device. Its characterization is given by means of the real time waveguide output intensity measurements, which correspond to the interaction between the sensing gold thin film window and the flowing analyte. The SPR curve was subsequently inferred. Finally, a modified version of the device is reported, with channel waveguides arranged in a Y-junction optical circuit, so that laser source stability requirements are lowered by a factor of 85 dB, making possible the use of low cost sources in practical applications.

## 1. Introduction

In the field of diagnostics, novel sensors have been designed in order to find the presence of viruses, bacteria, molecules and -in general- biological and chemical substances. These devices (biosensors), are biochemical-physical systems, in which biological mediators, also living, are immobilized according to particular protocols and coupled to suitable signal transducers able to record, selectively and reversibly, the concentration or the activity of various analytes [1].

Biosensors are used in many applications [2, 3], since they show low cost, ability to work *in situ* and scientific reliability. Surface plasmon resonance-based (SPR) sensors are sensitive and reliable optical biosensors [4]. The importance of this optical phenomenon consists – at present – in the possibility of surveying and identifying biological and chemical substances in many fields: medical diagnosis, environment monitoring, alimentary emergency and hygiene. The SPR technique constitutes a valid alternative to more traditional methods of analyses that demand long times and excessive costs. Competing research solutions are based on other optical techniques such as interferometry, fluorescence spectroscopy, and various guided-wave configurations [5 and references therein].

The SPR technique can be used for a wide range of biotechnological applications, including immunological analysis, studies of protein-protein interaction, molecular-biological studies on the mechanism of gene expression, signal transduction and cell-cell interactions, screening of new ligands, quantification of protein adsorption and immobilization, evaluation of surfaces for biocompatibility, determination of affinity constants, and examination of binding kinetics [6].

In this article, results of real time SPR interaction between optical guided waves [7] and different solutions with refractive indexes in the range of interest (1.3÷1.5) will be discussed, but the same configuration can be adapted to other values by a proper choice of the substrate material or with the introduction of suitable overlayers [8]. Measurements have been done using the configuration where an optical channel waveguide (on glass substrates) has a suitable portion –the interaction window–covered with a Cr/Au thin layer; a flow cell over the interaction window has been used for the fluid circulation set-up; the analytes were mixtures (with different percentages) of glycerol and water. Optimum gold film window thickness and width in this guided-wave configuration has been derived for the first time and implemented on a prototype device.

To improve sensitivity and lower laser source stability requirements, this setup has been modified with channel waveguides arranged in a Y-junction optical circuit, which allows the real time measurement of the output ratio between the reference and SPR branches, so that “common mode” intensity fluctuations are highly attenuated [9], making possible the use of low cost sources in practical applications.

## 2. Surface Plasmon Resonance

**Figure 1.**
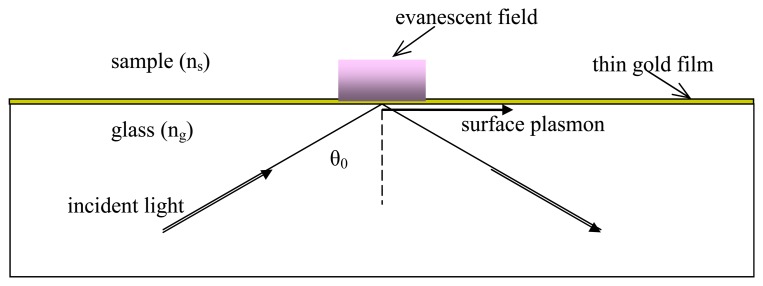
Basic setup for the excitation of surface plasmons by means of a light beam.

The surface plasmon is a well-known phenomenon of charge density waves occurring at the interface between a metal and a dielectric [10]. A surface plasmon can be in particular excited by collimated and TM polarized light beams with the basic configuration illustrated in Figure 1, where a gold thin film is deposited on a glass slide and a liquid sample material (analyte) covers the beam interaction region. Actually, a plasmon, and its associated evanescent field, appear at the upper metal interface for a specific value of the incident angle θ_0_, called *resonant angle*, which for a given setup depends on sample (i.e., cover material) refractive index n_s_.

As a practical matter, if we monitor instead the intensity distribution of the reflected light under focused beam illumination, using e.g. array detectors, we will find a sharp dip at a position corresponding to the resonant angle. This selective energy transfer is a consequence of the interaction between photons of the incident light beam and quasi-free electrons on the surface of the metal: when conditions of energy and momentum matching are satisfied, the photons can effectively couple with surface electrons creating a collective charge wave, whose macroscopic counterpart is an electromagnetic wave called surface plasmon wave (SPW). This interaction causes the selective reduction of the intensity of the reflected light. The curve, that expresses the dependency of the intensity of the reflected light from the angle of incidence θ, with which the light hits the interface, is called SPR curve and the θ_0_ angle is defined as the angle of surface plasmon resonance.

Fortunately, to model a practical device our phenomenon can be described entirely in classical terms as follows: an evanescent field is generated when the incident light beam undergoes total internal reflection at the glass-metal interface; this evanescent field then penetrates into the metal film and propagates to the upper metal surface with exponentially decreasing amplitudes. If the film thickness is not greater than a few hundreds Å, the evanescent field can interact with a covering layer (analyte), so that biomolecular interactions can be detected as they determine changes in the analyte layer thickness and refractive index [11].

From this last point of view, resonance occurs when the horizontal projection of the propagation vector of the optical wave is equal to the real part of the propagation factor of the SPW. If K_g,//_ and K_sp,re_ are, respectively, the horizontal projection of the incident light propagation vector (direction given by angle θ) and the propagation factor of the SPW, then the following equality must take place:

(1) $$k_{sp,re}=k_{0}{\left(\frac{{ɛ}_{m,re}{ɛ}_{s}}{{ɛ}_{m,re}+{ɛ}_{s}}\right)}^{\overset{1}{\;}/\underset{2}{\;}}=k_{g,//}=\frac{\omega }{c}\sqrt{{ɛ}_{g}}\sin\vartheta ,$$

where ε_g_ denotes the dielectric constant of glass, and ε_m,re_ the real part of the complex dielectric constant of metal.

From the last resonance expression, we can at first observe that possible solution frequencies will depend on the incident angle and also on the refractive indexes of the metal and of the dielectric substrate. Therefore, in a practical situation with a fixed wavelength light source, the condition of SPR could be verified for just one optical radiation incidence angle, as the dielectric/metal and corresponding refractive indexes are known. Moreover, a straightforward electromagnetic analysis shows that for resonance to exist light must be TM polarized and the two media must have dielectric constants of opposite sign. This condition can be practically obtained if one of two media is a metal: in fact, metal dielectric constant is function of the wavelength and in particular it assumes negative values in the region of the spectrum that ranges from the infrared to the visible. The geometrical setup, described in Figure 1, is the well known bulk Kretschmann configuration. Such a configuration is eventually strictly necessary, since, from Maxell's equations, it can be easily deduced that it is not possible to excite SPWs by a simple direct illuminating system [12].

In summary, the surface plasmon resonance angle depends on, mainly, the properties of the metal film, the wavelength of the incident light and the refractive index of the medium on the upper side of the metal film. The metal must have conduction band electrons able to resonate with the incoming light at a suitable wavelength. Metals that satisfy this condition are silver, gold, copper, aluminum, sodium and indium. However gold is the first choice as it is very resistant to oxidation and other atmospheric contaminants and it is compatible with a lot of chemical modification systems [13].

The thickness of the thin film of the metal is typically in bulk configuration about 40-50 nm: such value is necessary in order to have a deep absorption peak and a tightened SPR curve. As will be reported in the next section, for our guided-wave configuration we found that the optimum value is instead around 19 nm for gold. Considering that an adhesion Cr layer of a few nm is required, we will see that values greater than the total value of 25 nm, lead rapidly to an SPR curve with a rather low sensitivity; instead, smaller values determine a slower reduction of the sensitivity itself [14].

## 3. SPR in Channel Waveguides

The same SPR interactions can be thus obtained conveniently in guided-wave configurations, using optical waveguides on different materials [15]. In order to design SPR-based optical devices, a proper choice of the substrates is necessary. From the analysis with our optical simulation tools (FIMMWAVE from Photon Design, BeamPROP from RSoft, custom MatLab code), low refractive index borosilicate glasses (values around 1.47 at 633 nm wavelength) were found to be a better choice than the more common soda-lime glass, when – as in our case – the goal is to determine the presence of contaminants in aqueous solutions, whose refractive indexes are around 1.33. In practice, it is necessary that the SPR absorption peak is positioned close enough to the index region we want to measure, and in turn the peak position can be lowered using a lower-index substrate.

The above reasons led us to choose SCHOTT's Borofloat^®^ 33 slides, 0.7 mm thick, which in addition to the low refractive index exhibit very good overall optical quality. Substrates refractive index was checked using the prism coupling technique with a polarized HeNe laser source, obtaining a value of 1.4701 (estimated error of +/-0.0002).

**Figure 2.**
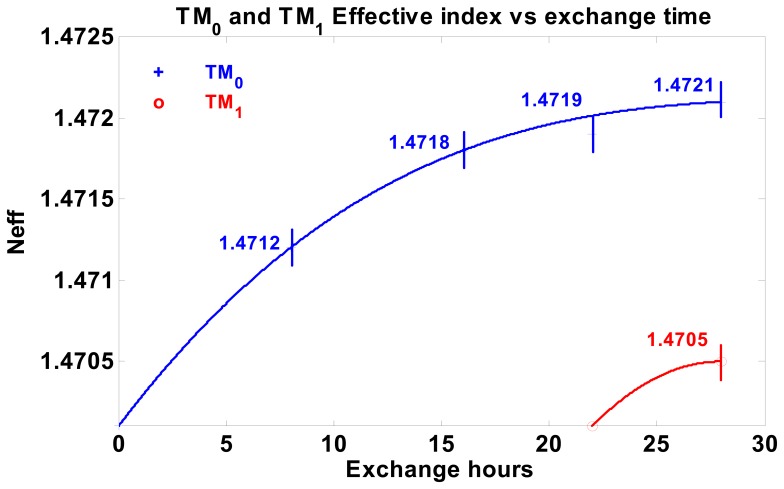
Experimental variation of waveguide mode effective indexes, N_eff_, as a function of exchange hours. Exchange carried out in fused KNO_3_ at 400 °C.

For waveguide fabrication, considering the relatively low Na content of this glass, we adopted KNO_3_ exchange at 400 °C, which gives a faster K^+^ ↔ Na^+^ exchange process in comparison to different molten ion sources [16]. In such a way, monomodal waveguides with optical losses lower than 0.1 dB/cm can be typically obtained in 8 hours, and modal effective indexes can also be varied using different exchange times, as shown in Figure 2, which contains measurements on several different planar waveguides fabricated with increasing exchange time, up to the appearance of the second guided mode.

Once found the correct ion exchange process conditions, we could proceed to the fabrication of the channel geometries required in a practical device: the 2D confined light in a channel waveguide can, with respect to bulk SPR, avoid the need of additional focusing optics and enhance the integration limit of the device; of course, the singlemode condition needs to be guaranteed to get better effect [17].

**Figure 3.**
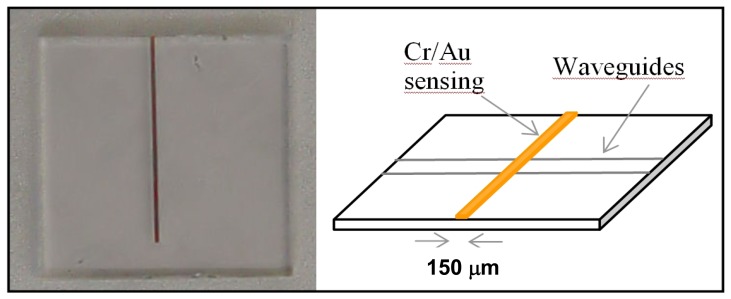
Photo and schematic of the glass sample region around the covering gold sensing window (channel waveguide is not visible, and this makes light coupling difficult).

To produce channel waveguides, a 1,000 Å thick Ni-Cr mask has been evaporated on the substrate and standard photolithography has been used to reproduce the waveguides pattern (array of channels from 2 to 18 μm) of our mask on the Ni-Cr metal film. We have eventually chosen a 16 hour exchange as the optimal process duration for the integrated waveguides. In Figure 3 a sample with integrated waveguides and a 25 nm sensing window (6 nm Cr film adhesion layer plus 19 nm gold layer) obtained by lift-off is illustrated.

**Figure 4.**
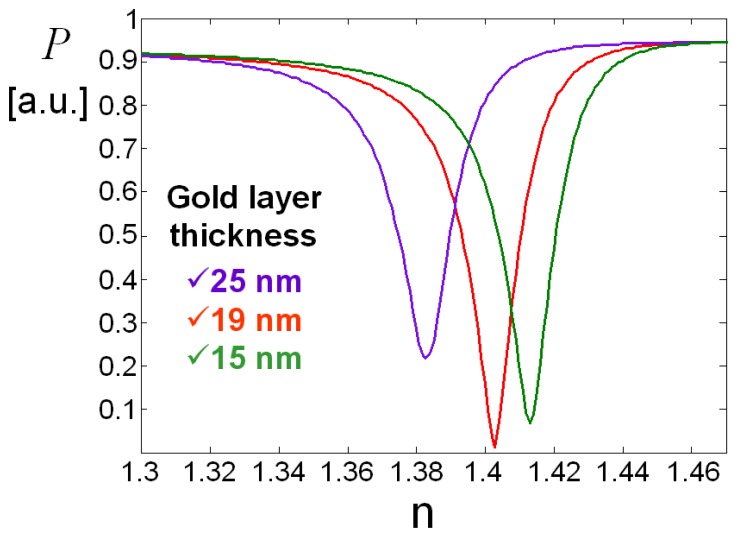
Resonance behavior vs. sensing window gold layer thickness (P, transmitted intensity; n, refractive index).

**Figure 5.**
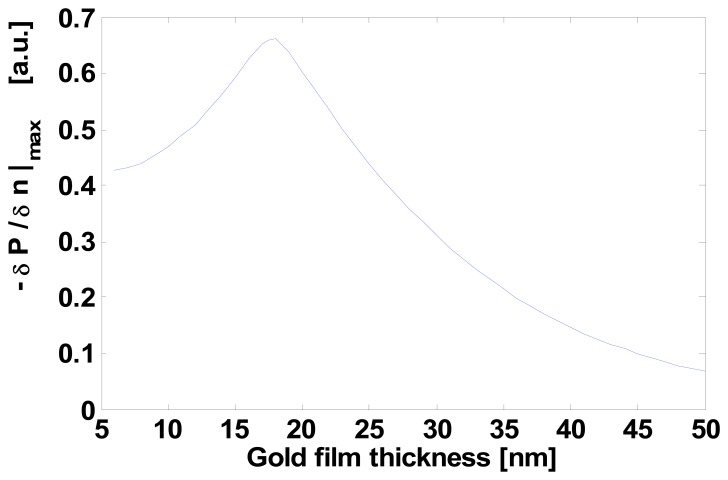
Power dependence (slope) from refractive index as a function of gold layer thickness (Cr film adhesion layer fixed at 6 nm).

The sensing metal window was just 150 μm in width (i.e., interaction length) in order to improve as much as possible the sensitivity and reduce overall losses. To find these optimal window thickness and width values, we investigated in detail the role of the two parameters using the numerical techniques implemented in an optical waveguide mode solver. In Figure 4 we illustrate the calculated resonance behavior vs. the sensing window gold layer thickness, whereas in Figure 5 we trace the power dependence (slope) from refractive index as a function of gold thickness: from this curve we obtained the optimum gold thin film thickness of about 19 nm, which gives the maximum device sensitivity in the chosen integrated optics configuration.

Finally, once the optimum sensing window thickness was found, its width was chosen by finding, with similar calculations, the value which maximizes the dynamic range offered by the best resonance curve of Figure 4, i.e. 150 μm. The resonant peak position fine tuning, can be obtained by trading-off some sensitivity and dynamic range.

## 4. Characterization of the device

**Figure 6.**
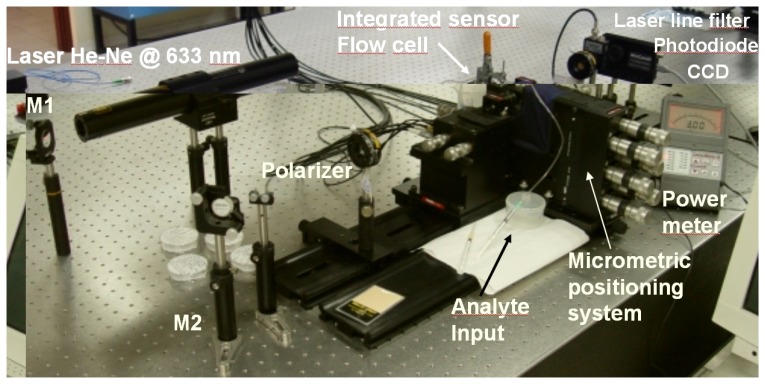
Waveguide coupling (with micropositioners) and measurement setup.

Optical characterization of the integrated sensor has been carried out using a polarized and stabilized HeNe laser and a calibrated power meter to measure the response intensity output of the waveguide with respect to the analyte refractive index. In Figure 6 the measurement set-up is pictured.

**Figure 7.**
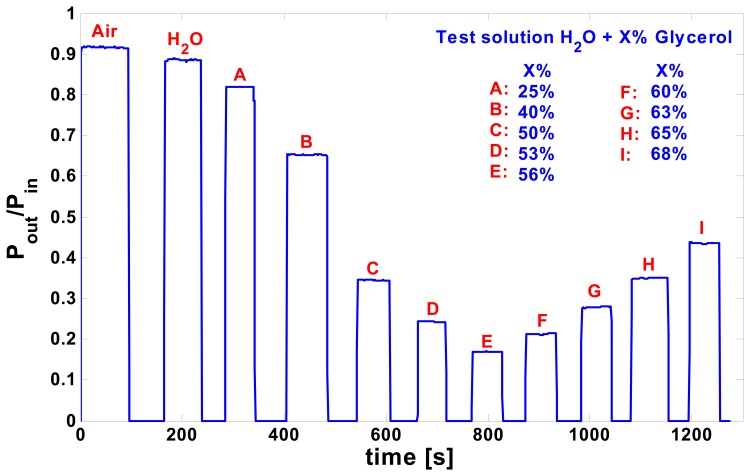
Real time waveguide normalized output intensity (P_out_/P_in_) measurements with different flowing test solutions (Water + Glycerol).

Considering that with a laser source at 632.8 nm wavelength, the maximum sensitivity was expected for refractive index values around 1.39, to characterize the device covering the required index range we used different solutions of glycerol and water (with percentages of glycerol varying between 0 and 70 %). The real time waveguide output intensity measurements, corresponding to the interaction between the sensing window and the different flowing test solutions, are reported in Figure 7 (intensity “blanking zones” correspond to change of test solution and laser beam switch off).

**Figure 8.**
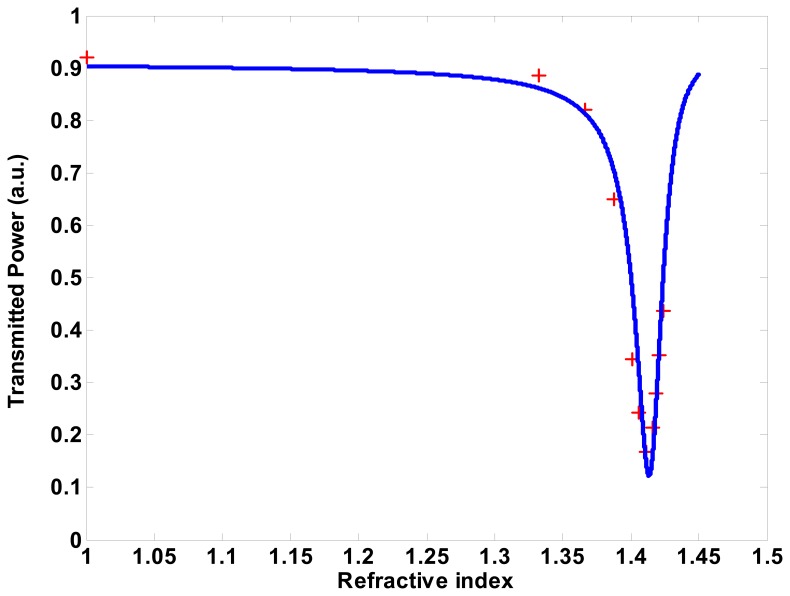
Normalized transmitted power (P/P_0_) vs. analyte refractive index n (i.e., % of glycerol) + points measured values; continuous line theoretical fitting.

Additionally, in Figure 8 the experimental data taken during the measurement in the more usual form of an SPR curve, together with a theoretical fitting which shows a very good agreement between design and experiment are reported.

Transmitted power, with respect to percentage of glycerol has been given. From this data, best sensitivity (@ 1.405 RI) is around 4,000 % RIU^-1^ (as customary in SPR literature, we write here RI and RIU for, respectively, Refractive Index and Refractive Index Unit), a value slightly higher than what found, for example, in the review paper [18]. Also, the resonance behavior of the best samples showed a good linearity in the range of 1.38 ÷ 1.41 RIU, a sensitivity of 0.2 μW / 100 μRIU, and a system resolution of 200 μRIU.

**Figure 9.**
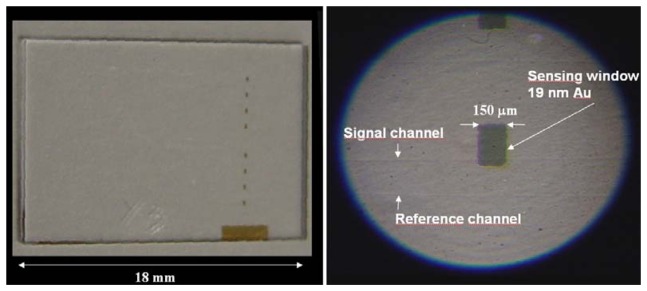
Photo and detail of the Y-junction device.

**Figure 10.**
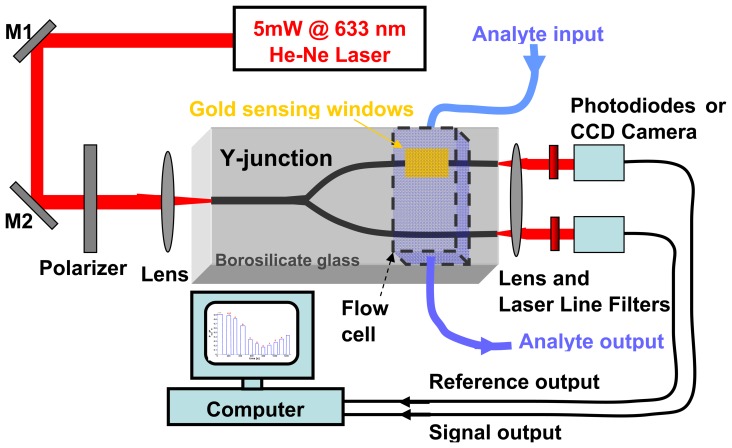
Measurement setup for the Y-junction device.

Finally, to conclude the report of our experimentation, we want to mention briefly a technical improvement that was introduced in the device to increase sensitivity and lower laser source stability requirements. More precisely, the device setup has been modified with channel waveguides arranged in a Y-junction optical circuit, following the approach adopted, for example, in [8]. A photo of the modified device in given in Figure 9, and the schematic of the new measurement setup in Figure 10.

**Figure 11.**
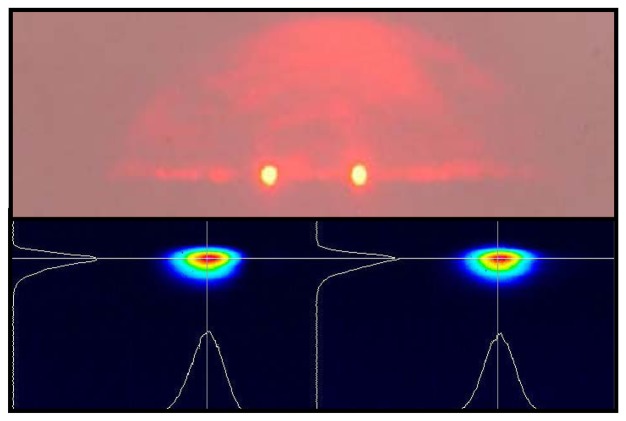
Output face of the Y-junction device: upper, picture of the spot images; lower, picture of the intensity maps obtained with a beam profiler (the curves correspond to the intensity along the superimposed horizontal and vertical lines passing from spot centers).

The Y-junction was characterized with the same laser source used for the prototype device and with an electronic circuit for the real time measurement of the output ratio between the reference and SPR branches (a picture of the output face, with the two laser spots images and intensity maps, is given in Figure 11), finding a 85 dB rejection of “common mode” measurements fluctuations.

## 5. Conclusions

We have fabricated and characterized optical waveguides ranging from 2 to 18 μm with optimum gold sensing windows on a borosilicate glass specific for index measurement in the range 1.3÷1.5. Best sensitivity (@ 1.405 RI) was around 4,000 % RIU^-1^. Also, the resonance behavior of the best samples showed a good linearity in the range of 1.38 ÷ 1.41 RIU, a sensitivity of 0.2 μW/100 μRIU, and a system resolution of 200 μRIU. A modified version of the device with channel waveguides arranged in a Y-junction optical circuit, allowed an 85 dB rejection of laser fluctuations, thus making possible the use of low cost laser sources for practical applications.
